# Multiple Sparse Representations Classification

**DOI:** 10.1371/journal.pone.0131968

**Published:** 2015-07-15

**Authors:** Esben Plenge, Stefan S. Klein, Wiro J. Niessen, Erik Meijering

**Affiliations:** 1 Biomedical Imaging Group Rotterdam, Departments of Medical Informatics and Radiology, Erasmus University Medical Center, Rotterdam, the Netherlands; National Institute of Genomic Medicine, MEXICO

## Abstract

Sparse representations classification (SRC) is a powerful technique for pixelwise classification of images and it is increasingly being used for a wide variety of image analysis tasks. The method uses sparse representation and learned redundant dictionaries to classify image pixels. In this empirical study we propose to further leverage the redundancy of the learned dictionaries to achieve a more accurate classifier. In conventional SRC, each image pixel is associated with a small patch surrounding it. Using these patches, a dictionary is trained for each class in a supervised fashion. Commonly, redundant/overcomplete dictionaries are trained and image patches are sparsely represented by a linear combination of only a few of the dictionary elements. Given a set of trained dictionaries, a new patch is sparse coded using each of them, and subsequently assigned to the class whose dictionary yields the minimum residual energy. We propose a generalization of this scheme. The method, which we call *multiple sparse representations classification* (mSRC), is based on the observation that an overcomplete, class specific dictionary is capable of generating multiple accurate and independent estimates of a patch belonging to the class. So instead of finding a single sparse representation of a patch for each dictionary, we find multiple, and the corresponding residual energies provides an enhanced statistic which is used to improve classification. We demonstrate the efficacy of mSRC for three example applications: pixelwise classification of texture images, lumen segmentation in carotid artery magnetic resonance imaging (MRI), and bifurcation point detection in carotid artery MRI. We compare our method with conventional SRC, *K*-nearest neighbor, and support vector machine classifiers. The results show that mSRC outperforms SRC and the other reference methods. In addition, we present an extensive evaluation of the effect of the main mSRC parameters: patch size, dictionary size, and sparsity level.

## Introduction

The use of learned, overcomplete dictionaries and sparse representations of signals has been shown to yield state-of-the-art performance in reconstructive image processing tasks [1–4], and in recent years the methodology has found application in image analysis as well [5–8]. Sparse representations can be applied because small-scale structures tend to repeat themselves throughout an image or a group of similar images. In the context of digital images, this phenomenon is also known as patch recurrence. Texture images, for instance, by definition have a high level of patch recurrence and they often have a very accurate sparse representation by dictionaries trained on similar images [9]. Natural images are generally rich in texture and other repeating structures and are abundant with patch recurrences, even across scales [10]. The bulk of studies on dictionary learning and sparse representations have therefore considered this type of images.

**Fig 1:**
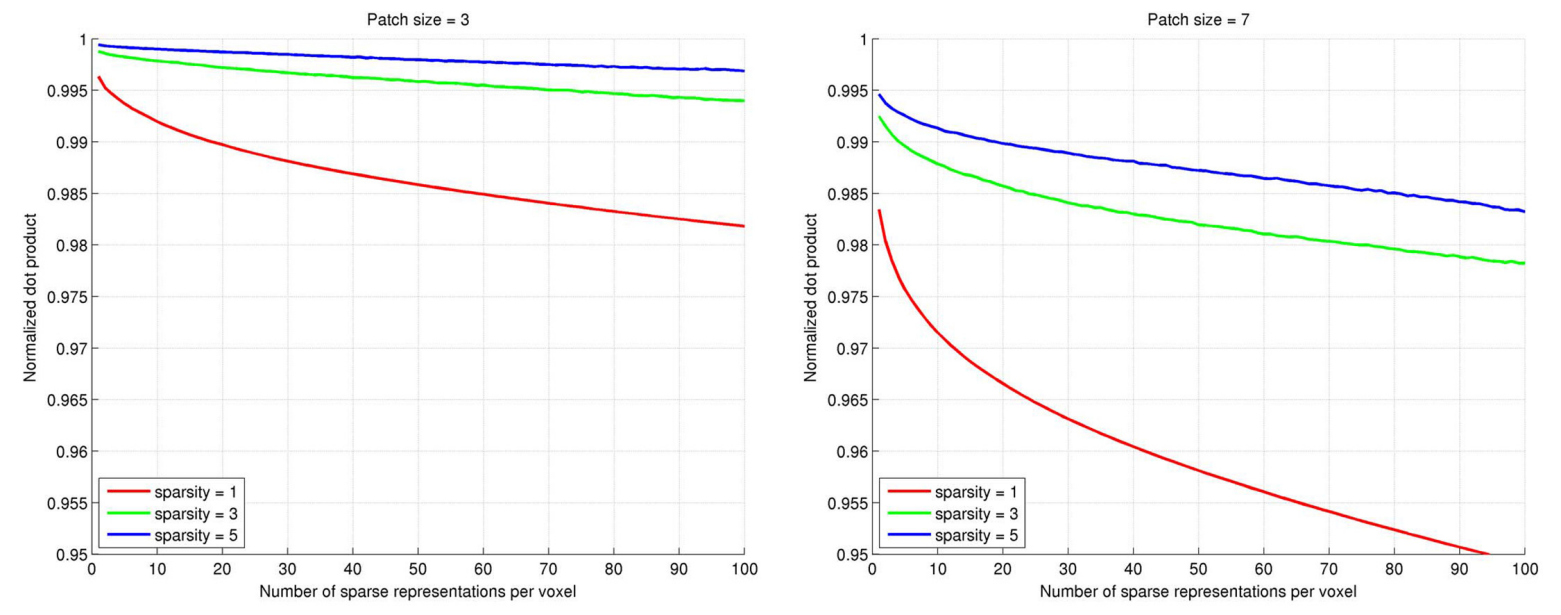
Illustration of the redundancy of dictionaries trained on a carotid MRI data set. We considered sparsity levels of 1 (red), 3 (green), and 5 atoms (blue curves), and patch sizes of 3 × 3 × 3 voxels (left) and 7 × 7 × 7 voxels (right), and trained a separate dictionary (1000 atoms) for each case. Approximately 1000 patches were then sampled randomly from a test image (a carotid MRI not included in the training set) and represented using each dictionary. The graphs show the average correlation of the sampled patches with their first 100 disjoint sparse representations using the dictionaries.

Quite different images are produced in the medical imaging domain. These images tend to be piecewise smooth and often do not contain much texture. Patch recurrence is present, though. Consider, for example, three-dimensional (3D) magnetic resonance imaging (MRI) data of a brain or a vessel tree. Both within and between the slices, small structures are repeated, and local patches can be well approximated by patches elsewhere in the image or in other, similar images. This is illustrated in Fig 1. The plots show the average correlation between patches sampled from an MRI of the carotid arteries and the 100 best approximations of each patch using sparse representations and a dictionary trained on other carotid MRIs. Note that the 100 sets of active dictionary elements used to encode each patch are disjoint. Two observations can be made from Fig 1: 1) The high correlation values suggest that patches in a carotid MRI are indeed very well represented by a linear combination of dictionary elements, commonly referred to as *atoms*, of a dictionary trained on patches from other carotid MRIs. 2) The dictionary is sufficiently redundant to accurately represent the new patches *many times* with disjoint sets of atoms.

If one thinks of a dictionary as a distribution, then the sparse representation of a new signal, using the dictionary, can be seen as a sample from the distribution. If the new signal is similar to the training data, it will be well approximated by the dictionary and thus likely under the distribution. If, however, the signal is very different, its dictionary representation will be less accurate, and the signal less likely under the distribution. This reasoning naturally leads to the use of dictionaries for classification. Sparse representation classification (SRC) methods use dictionaries and sparse representations for classification. In the basic SRC scheme, a dictionary is learned for each signal class, and classification of a new signal is done by finding the class whose dictionary approximates the signal with the smallest residual energy, given some sparsity constraint.

In this paper we present a generalization of SRC based on the observations in Fig 1. We hypothesize that the combination of multiple class estimates yields higher accuracy than using a single one. So instead of using a single signal representation from the dictionary of each class, we use multiple disjoint sparse representations, and for each class we fuse the residual energies of the multiple signal approximations into a soft class assignment. We shall refer to this new approach as *multiple sparse representations classification* (mSRC). We investigate the effect of going from one to multiple sparse representations and study the interplay between the number of sparse representations used and the basic SRC parameters (patch size, dictionary size, and sparsity level). We demonstrate the method’s performance for three different applications: texture classification, carotid lumen segmentation in MRI, and carotid bifurcation detection in MRI. In all applications, we compare the performance with conventional SRC, *K*-nearest neighbor (*K*-NN), and support vector machine (SVM) classifiers.

## Previous Work

Early works in the field of SRC considered the problem of texture classification [11, 12]. Overcomplete dictionaries were trained for multiple types of textures, and classification of a new image sample was performed by associating it with the texture type whose dictionary resulted in the minimum residual error, essentially implementing the SRC idea described in the introduction. Such dictionaries are called reconstructive because they focus on the task of accurate representation, and they lead to a generative classifier.

Later, the concept of discriminative dictionaries was proposed with the notion that for classification purposes a dictionary should not only represent a certain class of structures well, but also represent structures from other classes poorly [5]. For training, a cost function was formulated over the dictionaries from all classes simultaneously. It included a discriminative softmax function penalizing a dictionary’s accurate representation of signals from the wrong class. The method was applied to texture classification and object detection [5]. Others have achieved discrimination by including the inter-dictionary coherence in the training cost function, promoting more distinct dictionaries [13].

A different approach, based on the use of a single dictionary, was proposed by researchers in face recognition [6]. There, the dictionary was not trained but consisted of many face images of a number of persons. A new face image was represented as a sparse linear combination of all atoms (faces) in the dictionary. Subsequently, the atoms used in the sparse representation were split into their respective classes, and the residual with respect to the input image was calculated per class. New input images were associated with the class that yielded the minimum residual.

Instead of basing classification on the representation residuals only, it has been proposed to take the sparse coding coefficients into account [14]. Using a structured dictionary, created by concatenating class-specific sub-dictionaries, the Fisher discrimination criterion can be imposed to decrease intra-class scatter and increase inter-class scatter of the coefficients of the training data. Classification can then be based on the reconstruction error as well as the distance to a mean coefficient vector learned during training. In [14], this was used for face, digit, and gender recognition.

In the context of MRI, sparse representation based methodology has been used mainly for image processing tasks such as denoising and super-resolution [2, 15, 16]. Recently, however, it has also been applied to MR image analysis, specifically image segmentation. One example is hippocampus and ventricle segmentation in brain MR data [17]. The method, inspired by the non-local means (NLM) method [18], is an adaptation of atlas-based segmentation to the local self-similarity paradigm, in which expert-annotated images are pre-registered to the target image and classification of a target voxel/patch is performed by weighting the labels of the most similar patches in a local neighborhood in the atlas images. It has been shown to work well for detection and grading of Alzheimer’s disease [19]. A similar approach has been applied to general brain structure segmentation [20].

Two alternative approaches to hippocampus segmentation in MRI rely on a registered set of atlas images and dictionaries extracted *for each voxel* in the target image [21]. In the first approach, all patches in a neighborhood around the target voxel position are extracted from the labeled training data and used directly as the voxel’s dictionary. After a target patch has been coded using the dictionary, it is assigned to the class whose contributing patches yield the minimal reconstruction error, much like in the approach of [6] described above. Here, sparse coding is performed using elastic nets [22]. In the second approach, discrimination is incorporated by adding a classification error term to the dictionary training objective function, thus training a dictionary and a classifier simultaneously. A target patch is classified by first representing the patch sparsely using the dictionary and then applying the classifier to the sparse coding coefficients.

SRC has also been applied to prostate segmentation in X-ray computed tomography (CT) images [23]. In a series of steps, involving feature selection among a number of context and appearance features, dictionary training by elastic nets [22], and a linear regression step that trains a soft classifier from the representation residuals, a discriminative classifier is created. Classification of a new sample is done over a number of iterations in which the classification results are refined by updates to the context features.

Over the years, the basic SRC idea [11] has been modified in various ways to improve its performance or adapt it to specific classification tasks. While this has been achieved, the complexity of the approaches have increased, and complicated processing pipelines have been built around the SRC to effectively perform the given tasks. In this work, we focus on how basic SRC can be improved by a generalization that takes the exploitation of the dictionary redundancy one step further. As such, the idea presented in the following can be applied to many of the methods described above.

## Materials and Methods

### Dictionary Learning

There are several ways to learn a dictionary [24]. Here we have adopted the K-SVD algorithm [25], which is a relatively efficient method that incorporates the sparsity prior in the training process. Let **X** = [**x**_1_ **x**_2_…**x**_*M*_] be a matrix of *L*_2_-normalized training signals **x**_*i*_ ∈ ℝ^*n*^. In this study, the latter are simply vectorized image patches describing the local neighborhood around the voxel in the patch center, where the size of the patches is chosen such that they capture the structures that are relevant for the task at hand. K-SVD attempts to solve the following problem:

$$\begin{array}{c} \underset{D,A}{\text{min}}{\left||X-DA|\right|}_{F}^{2}\;\text{s.t.}\;{\left|\right|a_{i}\left|\right|}_{0}\leq T\;\forall i, \end{array}$$ (1)

where **D** ∈ ℝ^*n* × *m*^ is the overcomplete dictionary to be learned, having *m* > *n* atoms (column vectors of the matrix), **A** = [**a**_1_ **a**_2_…**a**_*M*_] is the matrix of corresponding sparse coding vectors **a**_*i*_ ∈ ℝ^*m*^, and ∣∣⋅∣∣_*F*_ is the Frobenius norm. Sparsity *T* limits the number of non-zero coefficients in each **a**_*i*_. The problem Eq (1) is a combinatorial one and a solution is, in general, intractable. K-SVD, however, approximates the solution by alternating between a greedy sparse coding step using the current dictionary estimate, and a dictionary update step. This has been shown to converge to a dictionary that is very close to the optimal one [26].

### Sparse Coding

Given a dictionary, **D**, the goal in sparse coding is to represent a signal **x** as a linear combination $\text{x}=\text{D}\hat{\text{a}}$, where $\hat{\text{a}}$ is sparse. Specifically, we wish to solve:

$$\begin{array}{c} \hat{a}=\underset{a}{\text{argmin}}{\left||x-Da|\right|}_{2}\;\text{s.t.}\;{\left||a|\right|}_{0}\leq T, \end{array}$$ (2)

where *T* limits the number of atoms used. As in dictionary training, the solution to this system is in general intractable. Instead, a locally optimal solution can be found by a greedy approach, for example orthogonal matching pursuit [27].

### Sparse Representation Based Classification

In basic SRC, the central voxel of an image patch is classified according to how well the patch is represented by the class-specific dictionaries. After a dictionary **D**_*i*_ has been trained for each class *i* ∈ {1…*N*} of image content, classification of a new patch **x**_new_ can be performed by evaluating the representation errors:

$$\begin{array}{c} e_{i}\left(x_{\text{new}}\right)={\left|\right|x_{\text{new}}-D_{i}{\hat{a}}_{i}\left|\right|}_{2}^{2}\;\forall i, \end{array}$$ (3)

where ${\hat{\text{a}}}_{i}$ has been found using Eq (2). From the class errors *e*_*i*_ a pseudo-probability measure, *p*(*C*_*i*_), is computed, leading to the following class assignment rule:

$$\begin{array}{c} i^{*}=\underset{i}{\text{argmax}}\;p\left(C_{i}\right)\;\text{where}\;p\left(C_{i}\right)=\frac{1}{N-1}\frac{\sum_{j=1,j\neq i}^{N}e_{j}}{\sum_{j=1}^{N}e_{j}}. \end{array}$$ (4)

### Multiple Sparse Representations Classification

The generalization of SRC that we propose, follows from the intuitive idea that by fusing the residuals of multiple disjoint sparse representations for each dictionary, a more robust class estimate of a given signal can be obtained. A related idea was explored recently for denoising applications [28] in which a randomization was included in the solution of Eq (2) such that many (in the order of 1000) (probably) distinct solutions were obtained and merged into an improved estimator of the underlying signal. Here, we take the idea in another direction and apply it to classification, in particular detection and segmentation.

The residuals of the disjoint sparse representations of a signal **x**_new_ are obtained by iteratively solving Eq (3) for all classes *i*. After each iteration *j*, each dictionary **D**_*i*_ is updated by removing the atoms that are non-zero in the current sparse coding vector ${\hat{\text{a}}}_{i}$, that is, the atoms that are used in the *j*th sparse representation of **x**_new_. Using *L* iterations, the voxel corresponding to **x**_new_ is assigned to class

$$\begin{array}{c} i^{*}=\underset{i}{\text{argmax}}\sum_{j=1}^{L}p_{j}\left(C_{i}\right), \end{array}$$ (5)

where *p*_*j*_(*C*_*i*_) is the pseudo-probability of class *i* found in the *j*th iteration. As seen in Fig 1, the subsequent signal approximations, *j* > 1, are still highly accurate. We hypothesize that the accumulation of information in the pseudo-probability derived from these approximations will improve the classification.

## Experimental Design

SRC is a generic methodology that has been shown to work for a number of different applications, including texture classification [11], object detection [5], face recognition [6], and digit recognition [14]. For the evaluation of our mSRC approach we chose three applications. The first is texture classification using sample images from the Brodatz database [29]. The second is carotid artery lumen segmentation in MRI, where the centerline of the carotid is given. And the third is bifurcation point detection in carotid artery MRI. In the following subsections we describe the design of each of these experiments in detail.

Besides testing the effect of fusing multiple sparse representations for classification (mSRC), we performed a series of experiments in which we optimized the mSRC parameters for the given task. The main free parameters of the dictionary training process are the patch size, the sparsity level, and the size of the dictionary. We employed grid search strategies to find the optimal parameter settings using a data set dedicated to parameter tuning (not used in the final testing).

In each experiment, we compared mSRC with basic SRC, *K*-nearest neighbors (*K*-NN), and support vector machine (SVM) classifiers. The used SVM [30] applies a radial basis function kernel as this is most common. Also, it makes SVM a nonlinear classifier and in that way similar to the other methods. Three SVM parameters were optimized: patch size, the SVM parameter *C*, which weighs the significance of wrongly classified samples during training, and the parameter *γ*, which is a parameter of the radial basis function kernel. As recommended [30] we tested the following ranges: *C* ∈ {2^−5^, 2^−3^, …, 2^13^, 2^15^}, and *γ* ∈ {2^−15^, 2^−13^, …, 2^1^, 2^3^}, although in some cases the range was extended to improve performance. For *K*-NN, the patch size and the values of *K* were optimized for performance.

In the *K*-NN experiments the training set was the same as for mSRC, given below. In the SVM experiments, because of the time consuming training process, only 1,500 training samples per class were used for each experiment. These samples were picked from random locations in each class-specific part of the training images.

### Texture Classification

**Fig 2:**
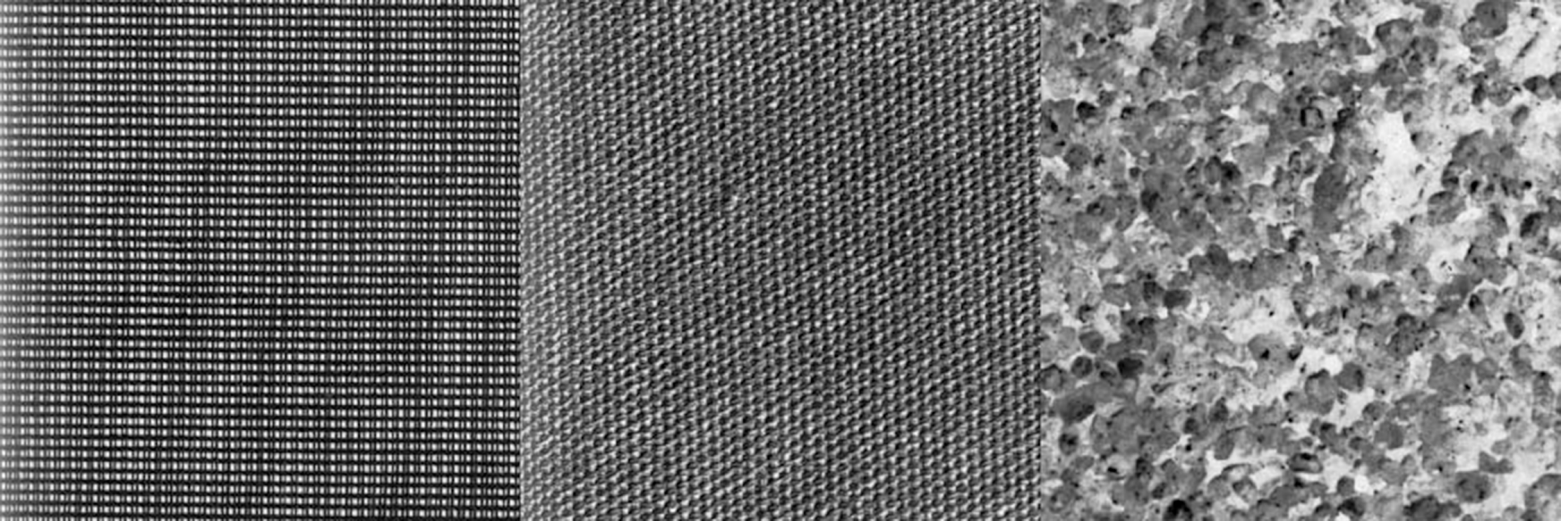
The three Brodatz textures used for the texture classification experiments. From left to right: D21, D77, D28.

The Brodatz texture data set has been used in a number of studies of SRC. For this study, three textures (D21, D28, and D77) were selected for a basic test of our hypothesis. D21 and D77 are textures of two types of cloth fabric, while D28 is a rock surface, see Fig 2. The texture images were split into a training part of 480 × 640 pixels and a (non-overlapping) test part of 160 × 640 pixels. Three pairwise classification experiments were carried out using the hit rate (the ratio of correctly classified pixels) as the performance measure. We investigated how the performance of mSRC varies with the number of sparse representations used. A dictionary size of 1000 atoms was used and a grid search optimization was performed on patch sizes of {3 × 3, 5 × 5, 7 × 7, 9 × 9, 11 × 11} pixels and sparsity levels of {1, 3, 5, 7, 9}. In each pairwise experiment, the size of the training set was approximately 300 000 samples per class.

### Carotid Artery Lumen Segmentation

**Fig 3:**
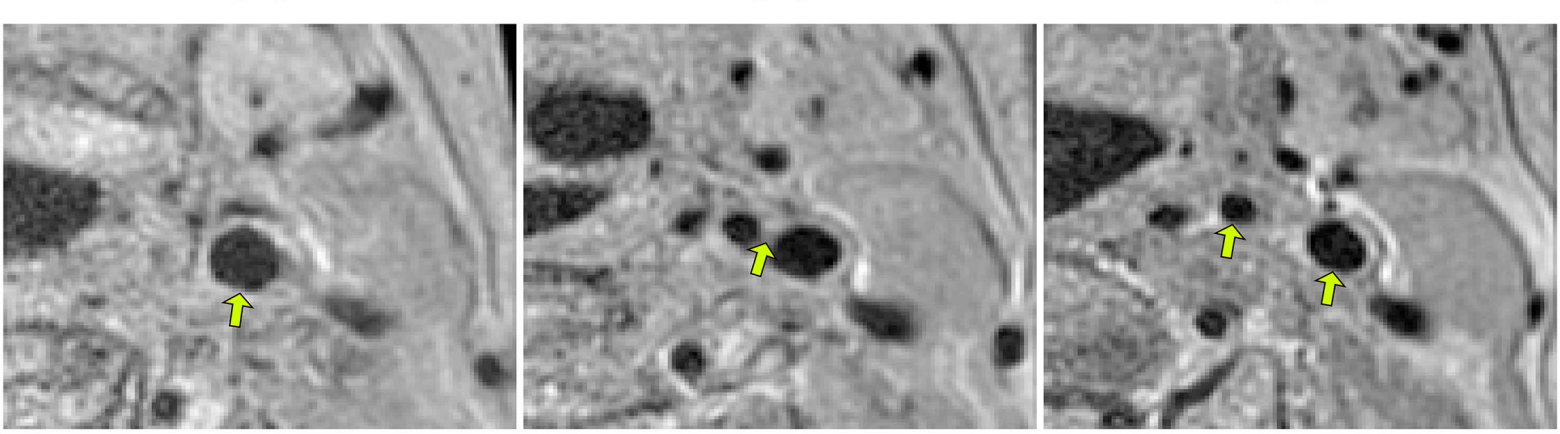
Carotid artery MRI. Cropped slices 10, 20, and 30 (left to right) of the left carotid of one subject in the carotid MRI data set. The arrows point to the common carotid artery (left image), the carotid bifurcation point (center image), and the internal and external parts of the carotid after the bifurcation (right image).

Segmentation of the carotid artery lumen is relevant in studies of atherosclerotic disease [31–33]. We posed the segmentation of the carotid arteries in MRI as a voxelwise classification problem, in which the carotid centerlines are given to guide the segmentation. Examples of cropped slices of a carotid artery MRI are shown in Fig 3. A data set consisting of 29 MR images of the head-and-neck region of 29 patients was used [33]. The data was anonymized prior to analysis and all patients gave written informed consent. The use of the data was approved by the Erasmus MC Medical Ethics Committee. The MRIs were proton-density weighted 2D spin echo images of resolution 0.5 × 0.5 × 0.9 mm, and size 256 × 256 × 51 voxels, acquired with a General Electric 1.5 T MRI scanner. For all 29 subjects, the left and right carotid centerlines and the lumens were annotated by experts. The images were bias corrected using N4ITK [34] and split into two sets: one used for parameter tuning and the other for the final experiment using the found optimal parameters. The former consisted of 8 images selected randomly from the full set of 29 images. Parameter tuning was performed by leave-one-out tests. The tuned parameters were patch size, sparsity level, and dictionary size. The other 21 images were partitioned into a training set consisting of 11 images and a test set consisting of the remaining 10 images.

In each experiment, the training data was organized by extracting cubic (in voxel space) patches centered on all voxels inside the annotated lumen regions of each training image. This resulted in approximately 14 000 lumen samples from each of the training images. A corresponding number of background patches were extracted from each training image at random locations outside the lumen but within a tubular region of interest (ROI) having a diameter of 31 voxels around the annotated centerline. All patches were vectorized and principle component analysis (PCA) was performed to reduce the dimensionality. Components with the lowest 0.1% of energy (60–84% of all, depending on the patch size) were discarded. Lumen and background dictionaries were trained on these signals.

Before segmentation using mSRC, each MRI image was split into a left and a right part, such that only a single carotid was present in each resulting image. Similar to the training phase, a tubular ROI was automatically generated around the annotated centerline. Such pre-processing is standard practice in carotid artery segmentation methods. At each voxel in the ROI, cubic patches were extracted and, after vectorization, projected into the lower-dimensionality space obtained by PCA during training. After assigning each voxel in the ROI to either foreground (carotid lumen) or background by mSRC, a simple 2D connected component procedure was performed: for each slice in the voxelwise classification image, a logic OR operation was performed with the corresponding slice of an inflated version of the centerline (diameter of 7 voxels). In each slice, only the connected component that included the inflated centerline was considered lumen in the final segmentation. Taking the annotated carotid artery lumen as ground truth, we used the Dice overlap score [35] as performance measure.

A two-stage parameter tuning process was performed for both SRC and mSRC. First, using a dictionary of 1000 atoms, a grid search optimization was performed on patch sizes of {3 × 3 × 3, 5 × 5 × 5, 7 × 7 × 7, 9 × 9 × 9, 11 × 11 × 11} voxels and sparsity levels of {1, 3, 5, 7, 9}. Next, the best performing subset of the patch sizes were selected for a 3D grid search in which the additional parameters were the sparsity levels, with the values listed above, and dictionaries with {200, 500, 1000, 2000, 4000} atoms.

### Carotid Artery Bifurcation Detection

Detection of the bifurcation in carotid artery MRI is useful for carotid centerline extraction, segmentation, and registration (in multimodal imaging or longitudinal studies). For this experiment the (bias corrected) MRI data set of 29 subject was partitioned into the same parameter tuning set and test set as described in the previous section. In all 29 subjects an expert annotated the bifurcation point and lumen of the left and right carotid arteries. An example of a bifurcation point can be seen at the arrow tip in the central image of Fig 3.

The bifurcation point dictionary was trained on image patches extracted in a region of 7 × 7 × 7 voxels centered on the annotated bifurcation points. To extend and introduce more variability into the training set, a number of rotations were applied to the training images, and at each rotation patches were extracted around the annotated (correspondingly rotated) bifurcation points and added to the bifurcation point training set. The set of angular rotations was {−30, −20, −10, 0,10, 20, 30} degrees and rotation was performed around the slice selection direction. This resulted in approximately 4700 samples from each of the training images. For each image, and each rotation, a corresponding number of patches were sampled at random locations in the rest of the image. Before training of the bifurcation point and background dictionaries, all patches were vectorized and PCA was performed to reduce the dimensionality. The components with the lowest 0.1% of energy (63–85% of all, depending on the patch size) were discarded.

As in the carotid artery segmentation experiments, the test images were split into their left and right halves, such that only a single bifurcation was present in each test image. Rectangular left and right ROIs were generated such that the annotated bifurcation points in all 29 images were contained in the ROIs. To reduce computation time, bifurcation point detection was performed inside the ROIs only. A class response image was generated by assigning a pseudo-probability to each voxel of it being the bifurcation point, and the voxel with the highest response was classified as the bifurcation point. While in SRC, mSRC, and SVM, class assignment is done by a continuous range of pseudo-probability values, in *K*-NN the soft class assignment is discretized into *K* levels. To decrease the likelihood of having multiple maximum response points, large values of *K* were used in the tuning, and a Gaussian filter with *σ* = 0.5 voxel was applied to the class response image. To make conditions uniform, this filter was applied to the response images of all four methods. Taking the annotated bifurcation points as ground truth, we used the distance (in millimeters) between the detected and annotated bifurcation points as performance measure.

A two-stage parameter optimization was performed for both SRC and mSRC. First, using a dictionary of 1000 atoms, a grid search optimization was performed on patch sizes of {3 × 3 × 3, 5 × 5 × 5, 7 × 7 × 7, 9 × 9 × 9, 11 × 11 × 11} voxels and sparsity levels of {1, 3, 5, 7}. Next, the best performing subset of patch sizes were selected for a 3D grid search in which the additional parameters were the sparsity levels, with the values listed above, and dictionary size, with values of {500, 1000, 2000, 4000} atoms.

## Results

In this section we present the results of the experiments and highlight the main observations. A more detailed discussion of the found effects is given in the next section.

### Texture Classification

**Fig 4:**
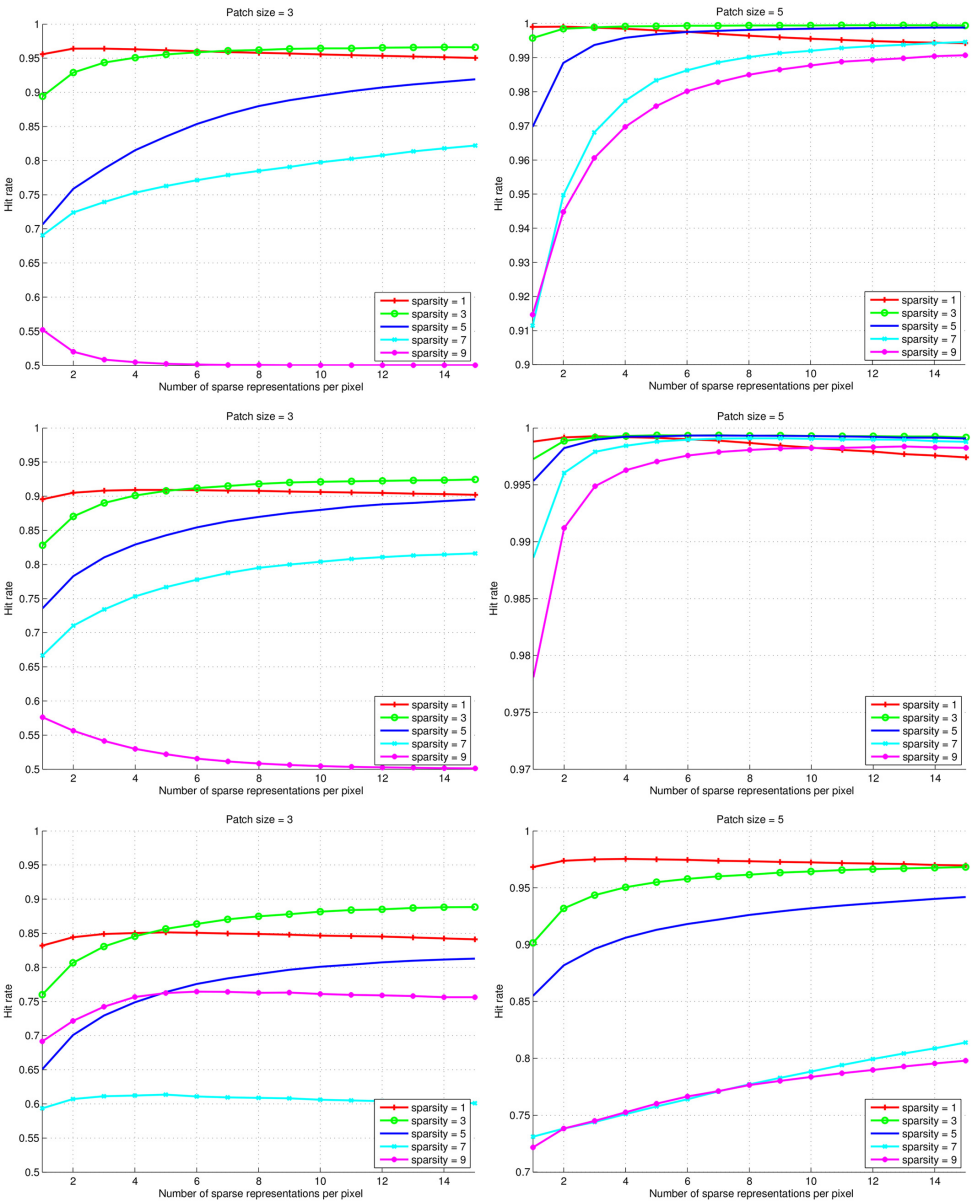
Results of the pairwise texture classification experiments. Top row: classification of textures D21 and D28. Middle row: classification of textures D21 and D77. Bottom row: classification of textures D28 and D77. Experiments with patch sizes of 3 × 3 and 5 × 5 pixels are shown in left and right columns, respectively. Note that the *y*-axes of the plots are scaled differently to allow appreciation of the effect of using multiple sparse representations.

The results of the pairwise texture classifications are shown for patch sizes 3 × 3 and 5 × 5 pixels and sparsity levels of {1, 3, 5, 7, 9} in Fig 4. For patch sizes of 9 × 9 pixels and larger, the results improve to near perfect classification (hit rates > 0.99). The positive effect of using mSRC is clear from the plots. The hit rates generally increase with the number of representations used. It can also be seen how the rate of improvement decreases with the number of representations. An exception is the results obtained with a sparsity level of 1: after an initial improvement in performance when using 2–4 sparse representations, a small but consistent decay is seen for higher numbers of sparse representations. Another exception to the positive effect of mSRC is seen in the top-left and mid-left plots, for sparsity levels of 9. Here, a clear negative effect of using multiple representations is found, and the hit rates move asymptotically towards 0.5; that is, a seemingly random class assignment is performed. In both cases, the hit rate is quite poor when using just a single sparse representation. A final observation from Fig 4 is that when only a single sparse representation is used, a sparsity level of 1 performs best. When using multiple sparse representation, higher sparsity levels generally perform better.

**Fig 5:**
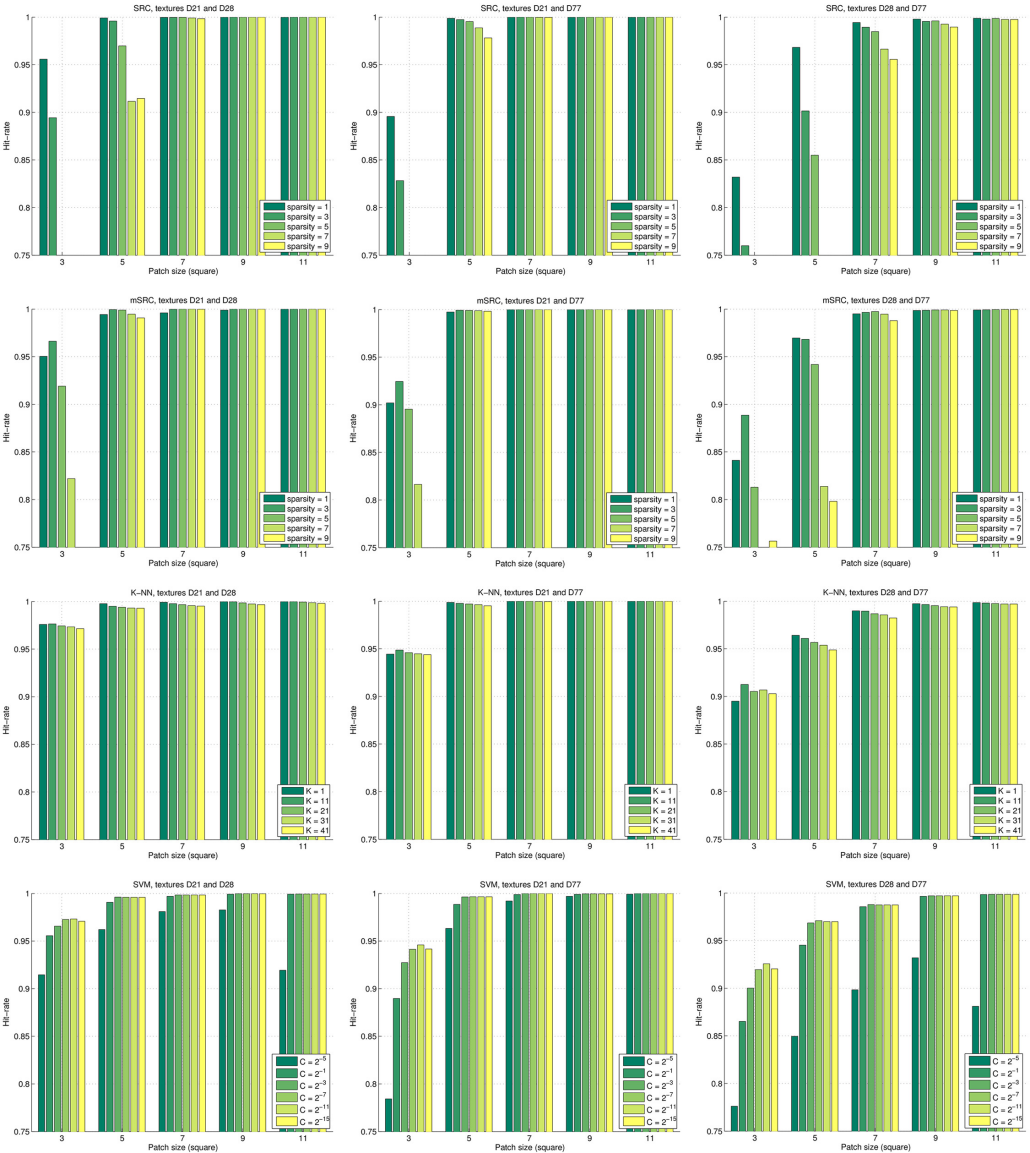
Comparison of the hit rates of four methods for texture classification. Top to bottom: results of SRC, mSRC (using 15 representations), *K*-NN, and SVM. Left to right: pairwise texture classification of D21 and D28, D21 and D77, and D28 and D77.

In Fig 5 the performance of mSRC using 15 representations is compared with that of basic SRC (for different sparsity levels), *K*-NN (for different values of *K*), and SVM (for different values of *C*) for the pairwise texture classifications. For SVM, parameters *C* and *γ* were both explored, but for visualization purposes only the SVM results with the optimal *γ* = 2^−19^ are included in the figure. The plots indicate that for small patch sizes, the performance of SRC and mSRC is generally inferior to that of *K*-NN and SVM, but for larger patch sizes, all methods perform very well. SRC with a sparsity level of 1 generally performs almost as good as the highest scoring sparsity level of mSRC. For sparsity levels larger than 1, mSRC consistently outperforms SRC.

### Carotid Artery Lumen Segmentation

**Fig 6:**
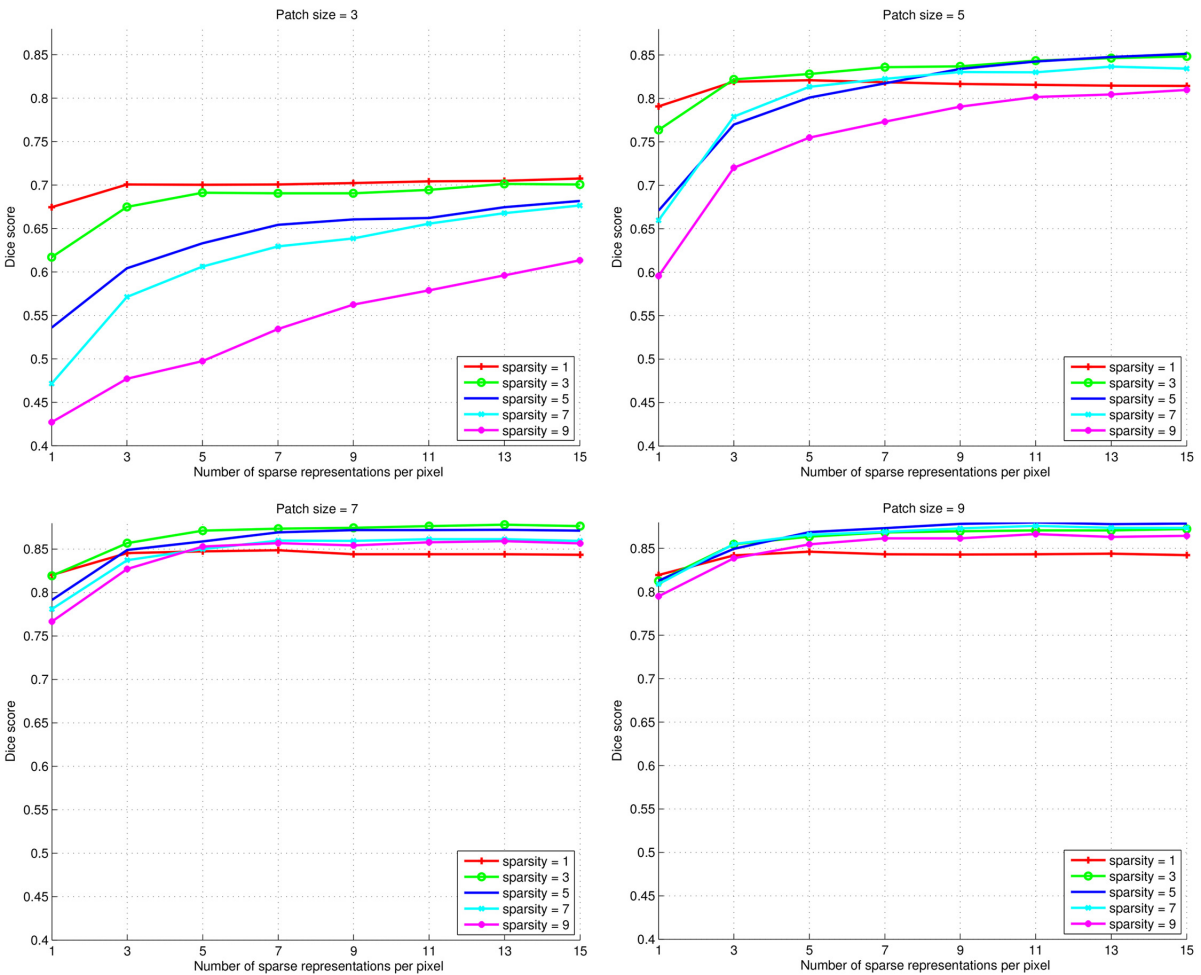
Dice scores in carotid artery lumen segmentation experiments for parameter tuning. The plots show (in scan-line order) experiments with patch sizes of {3 × 3 × 3, 5 × 5 × 5, 7 × 7 × 7, 9 × 9 × 9} voxels. In each sub-plot experiments with sparsity levels of {1, 3, 5, 7, 9} are included.

The results of parameter tuning for carotid artery lumen segmentation are shown in Fig 6. It can be seen how the segmentation performance generally improves with the number of sparse representations used, resembling the results of the texture experiments. As in the texture experiments, an exception to the trend occurs (in some of the cases) for sparsity levels of 1: after a small increase, the performance decreases slightly when using more sparse representations.

It is clear that the patch size has a significant impact on the segmentation performance, which increases when going from a patch size of 3 × 3 × 3 to 7 × 7 × 7 voxels. The highest Dice scores obtained with patch size 9 × 9 × 9 are slightly better than those of 7 × 7 × 7. Experiments were also performed with patch sizes of 11 × 11 × 11 voxels but the results of these were inferior to those of patch sizes of both 7 × 7 × 7 and 9 × 9 × 9 voxels and therefore are omitted here.

The effect of the sparsity level is clear for small numbers of sparse representations. When using only a single sparse representation, there is a consistent order to the performance of the sparsity levels: using 1 atom yields best performance, followed by using 3, 5, 7, and 9 atoms. Interestingly, this general order was also seen in the texture experiments. When more sparse representations are used (and when the patch size is larger than 3 × 3 × 3), sparsity levels of 3, 5, and 7 consistently outperform sparsity levels of 1 and 9.

**Fig 7:**
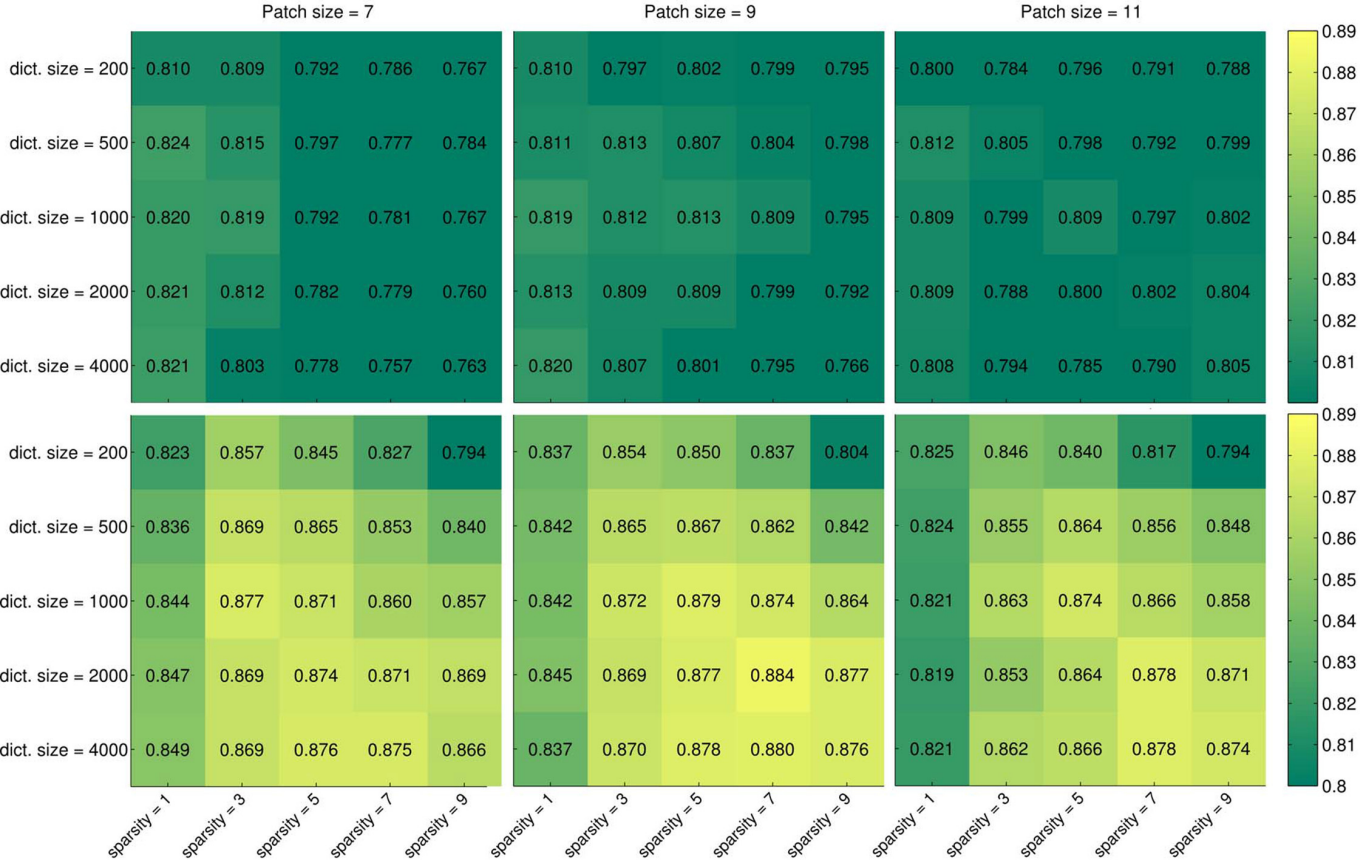
Heatmaps of carotid artery lumen segmentation results for parameter tuning. Numerical and color coded Dice scores are shown for dictionary sizes of {200, 500, 1000, 2000, 4000} atoms, patch sizes of {7 × 7 × 7, 9 × 9 × 9, 11 × 11 × 11} voxels, and sparsity levels of {1, 3, 5, 7, 9}. Top row: a single representation is used for classification. Bottom row: 15 representations are used for classification.

Further experiments were performed to examine the influence of the dictionary size on the performance. The results are visualized in Fig 7 for dictionaries of {200, 500, 1000, 2000, 4000} atoms using SRC and mSRC with 15 sparse representations. For mSRC, the performance of dictionaries with 2000 and 4000 atoms is very similar and only slightly better than when using 1000 atoms. The overall optimal parameters are a dictionary size of 2000 atoms, a patch size of 9 × 9 × 9 voxels, and a sparsity level of 7 atoms. These parameters were used for final testing.

The parameters of the reference methods were optimized as well. For SRC, the following parameters yielded the best Dice score: dictionary size of 500 atoms, patch size of 7 × 7 × 7 voxels, and sparsity level of 1. For *K*-NN, patches of size {3 × 3 × 3, 5 × 5 × 5, 7 × 7 × 7, 9 × 9 × 9, 11 × 11 × 11} voxels and *K*s of {1, 11, 21, 31, 41} were tested, and a patch size of 3 × 3 × 3 and a *K* of 31 were found to be best. For SVM, the same patch sizes were tested, and the optimal parameters were a patch size of 3 × 3 × 3, *C* = 2, and *γ* = 2^−19^.

**Fig 8:**
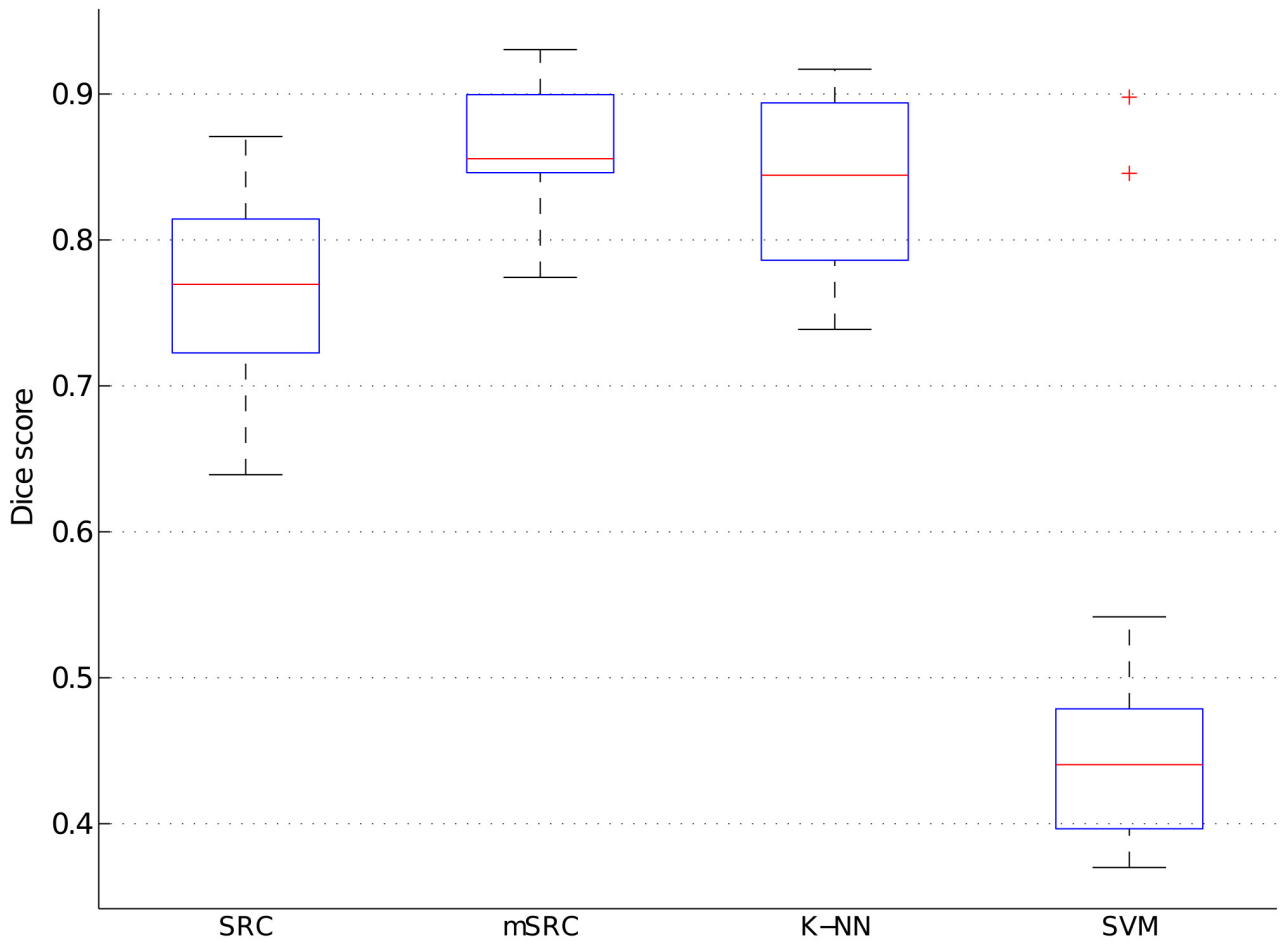
Performance of SRC, mSRC, *K*-NN, and SVM in the carotid artery lumen segmentation experiments. The results are shown as boxplots, where the blue box indicates the 25–75 percentile, the red bar indicates the median, the whiskers are determined by the default MATLAB boxplot settings (see MATLAB documentation), and the individual red plus-markers are outliers.

The results of the carotid artery lumen segmentation experiments on the final test set after parameter optimization are shown in Fig 8. The best performance, measured by the median Dice score, was achieved by mSRC (0.855), followed by *K*-NN (0.845), and SRC (0.779). SVM (0.440) performed considerably worse than the other methods.

### Carotid Artery Bifurcation Detection

**Fig 9:**
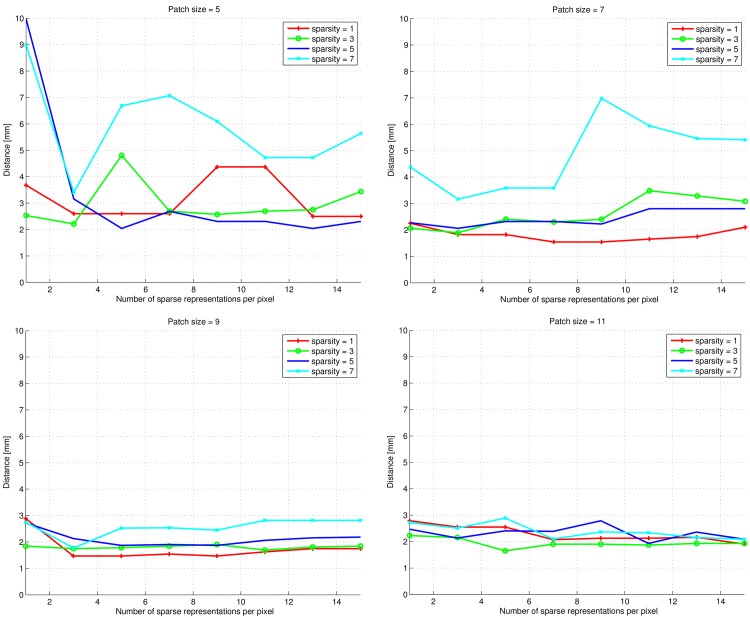
Euclidean distances (in mm) between true carotid artery bifurcation points and detected ones. Plots are shown for patch sizes of {5 × 5 × 5, 7 × 7 × 7, 9 × 9 × 9, 11 × 11 × 11} voxels and sparsity levels of {1, 3, 5, 7}.

The results of parameter tuning for carotid artery bifurcation detection are shown in Fig 9. Again, the patch size has a clear effect: for larger patch sizes, the bifurcation points are detected with smaller median distances to the ground truth points, and the seemingly erratic effect of the sparsity level is reduced. The effect of using multiple sparse representations is not clear. For patch sizes of 9 × 9 × 9 and 11 × 11 × 11 voxels, when using more than one sparse representation there is a tendency of decreased distance to the ground truth points, but in many cases this effect reverses when using a high number of representations. In Fig 9 the best overall result is seen to be obtained by a patch size of 9 × 9 × 9, a sparsity level of 1 and using nine sparse representations. In the lack of a clear trend we used these settings for further experiments.

**Fig 10:**
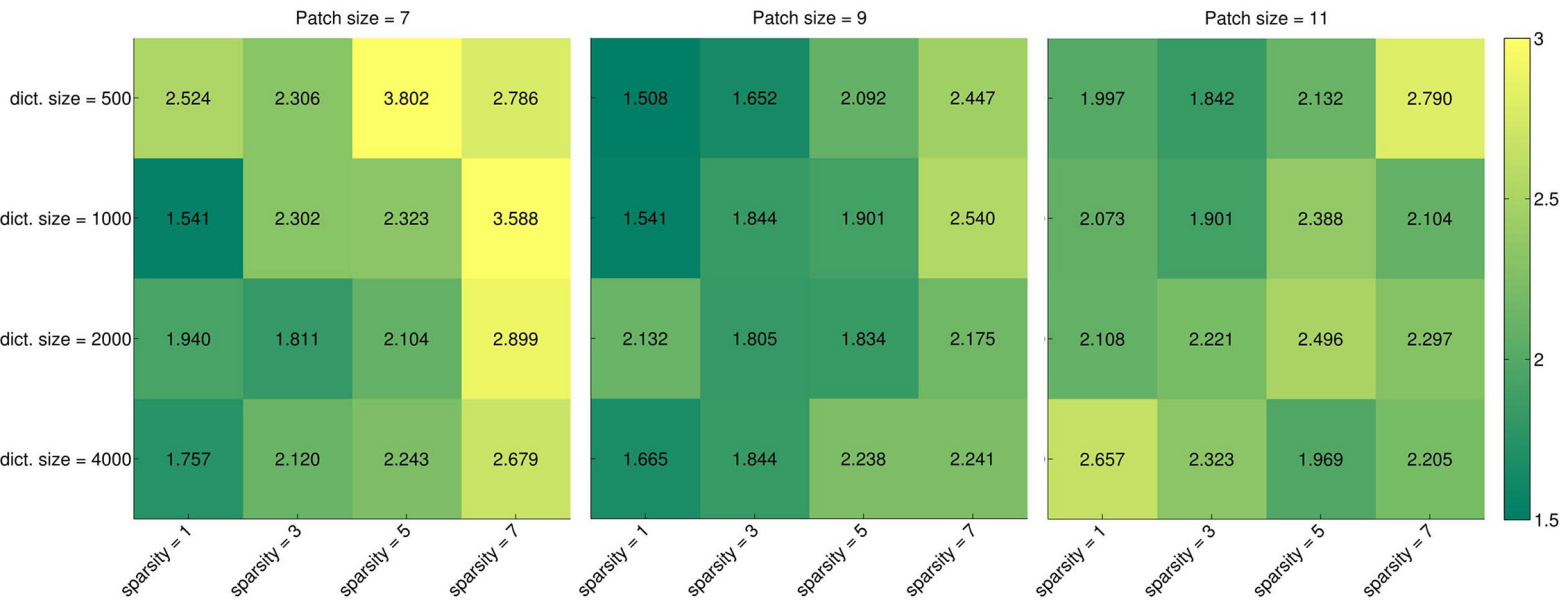
Heatmaps of carotid artery bifurcation detection results for parameter tuning. Numerical and color coded distances to the ground truth are shown for dictionary sizes of {500, 1000, 2000, 4000} atoms, patch sizes of {7 × 7 × 7, 9 × 9 × 9, 11 × 11 × 11} voxels, and sparsity levels of {1, 3, 5, 7}.

The heatmap visualization in Fig 10 shows the effect of dictionary size and sparsity level for given patch sizes. Again, it is hard to draw general conclusions about the effect of the parameter settings from these plots, but there is a tendency for lower sparsity levels to perform better. Based on Figs 9 and 10 we estimated the optimal settings for mSRC to be a dictionary size of 500 atoms, a patch size of 9 × 9 × 9 voxels, a sparsity level of 1, and nine sparse representations. These parameter values were used for mSRC bifurcation detection on the test set.

The parameters of the reference methods were optimized as well. For SRC, the same parameters were tested as for mSRC. The optimal values were found to be a dictionary size of 500 atoms, a patch size of 9 × 9 × 9 voxels, and a sparsity level of 7. For *K*-NN, patches of {3 × 3 × 3, 5 × 5 × 5, 7 × 7 × 7, 9 × 9 × 9, 11 × 11 × 11} voxels and *K*-values of {100, 200, 300, 400, 500} were tested, and a patch size of 5 × 5 × 5 voxels and a *K* of 200 were found to yield the best performance. For SVM, the same patch sizes were tested, and the optimal parameters were a patch size of 3 × 3 × 3 voxels, *C* = 8192, and *γ* = 2^−19^.

**Fig 11:**
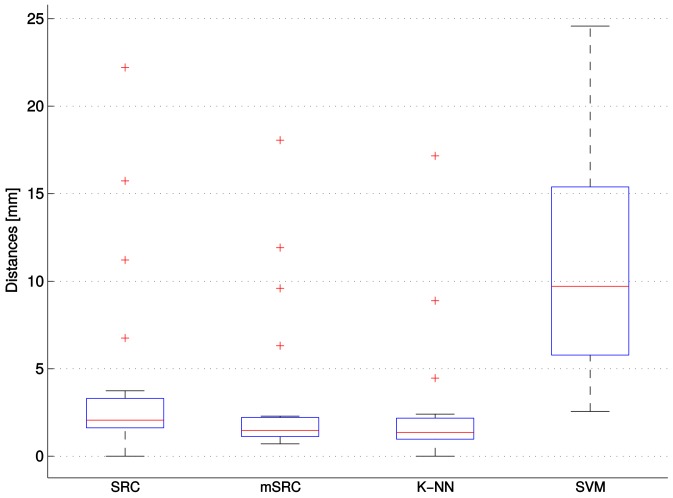
Performance of SRC, mSRC, *K*-NN, and SVM in the carotid artery bifurcation detection experiments. The results are shown as boxplots, where the blue box indicates the 25–75 percentile, the red bar indicates the median, the whiskers are determined by the default MATLAB boxplot settings (see MATLAB documentation), and the individual red plus-markers are outliers.

The results of the carotid artery bifurcation detection experiments on the final test set after parameter optimization are shown in Fig 11. The best performance, measured by the median distance of the detected bifurcation points to the ground truth, was achieved by *K*-NN (1.35 mm), followed by mSRC (1.46 mm), and SRC (2.06 mm). SVM (9.71 mm) performed considerably worse than the other methods.

## Discussion

### Effect of Number of Representations

From Figs 4 and 6 we conclude that using multiple sparse representations may considerably increase the performance of voxelwise classification, with the largest increase achieved in the first few additional representations. However, too low (level 1) or too high sparsity (levels larger than 7) may have a negative effect. This calls for careful selection of the sparsity level and number of representations for a given application. In the texture classification experiments (Figs 4 and 5) we observed little gain by using mSRC instead of SRC. For small patch sizes there was some improvement in performance but for larger patches both methods yielded near perfect classification. In fact, all tested methods (depending on the parameter settings) yielded near perfect classification in these experiments (Fig 5), so plain texture classification may be a too easy problem for an informative comparison of the methods. In the carotid artery lumen segmentation problem, on the other hand, we observed a clear difference in the performance of SRC and mSRC (Figs 6–8). While SRC performed reasonably with a sparsity level of 1, pronounced improvements in performance were achieved when using mSRC in combination with higher sparsity levels and patch sizes larger than 3 × 3 × 3 voxels. In the following we shall discuss how these parameters interact.

### Effect of Patch Size

The patch size has a significant impact on classification performance, not only for (m)SRC, but also for *K*-NN and SVM. This was to be expected, as it is the patch size that determines whether sufficient local information is captured to enable class discrimination. In the degenerate case of using a single voxel as the feature, no discriminative information is present (intensity information is discarded in (m)SRC due to the scaling factor in its generative model). However, the patch size can also become too large. If a patch captures more than the essential features of a class, its distance to other samples of the class increases and its properties do not generalize to discrimination. So, for every application, the patch size should be optimized against the scale of the structural components of interest and the image resolution.

As noted in the introduction, there is a qualitative difference between the highly textured natural images and the piecewise smooth medical images. The textures included in this study are highly repetitive, and a good descriptor should capture at least a few periods of such repetitions. In Fig 5 it is seen that a patch size of 5 × 5 or 7 × 7 pixels in the texture experiments was sufficient to capture the discriminative structures for both mSRC and the reference methods. Larger patches performed even better.

In the considered MR images, patch sizes of 7 × 7 × 7 and 9 × 9 × 9 voxels captured the discriminative structures well for (m)SRC, in both the carotid artery lumen segmentation and bifurcation point detection experiments. Interestingly, for this application *K*-NN and SVM performed better for smaller patch sizes (3 × 3 × 3 and 5 × 5 × 5) than for larger ones. The reason for this may be that both *K*-NN and SVM include the patch intensity in their class model, and that small patches are sufficient to capture the intensity.

### Effect of Sparsity Level

Given a dictionary size, the sparsity level determines the flexibility of the representation. The higher the sparsity level, the easier it becomes to approximate a given signal, be it from the dictionary’s class or not. One clear result observed from Figs 4 and 6, however, is that the flexibility provided by higher sparsity levels does not help in SRC (that is, mSRC using 1 representation): in all cases the classification or segmentation performance is ordered by sparsity level, with a sparsity level of 1 on top. The reason for this may be that higher sparsity levels can lead to over-fitting. In other words, the first few atoms included in the sparse representation encode the main components of the underlying signal of input patch. The last few atoms on the other hand, might only encode noise.

**Fig 12:**
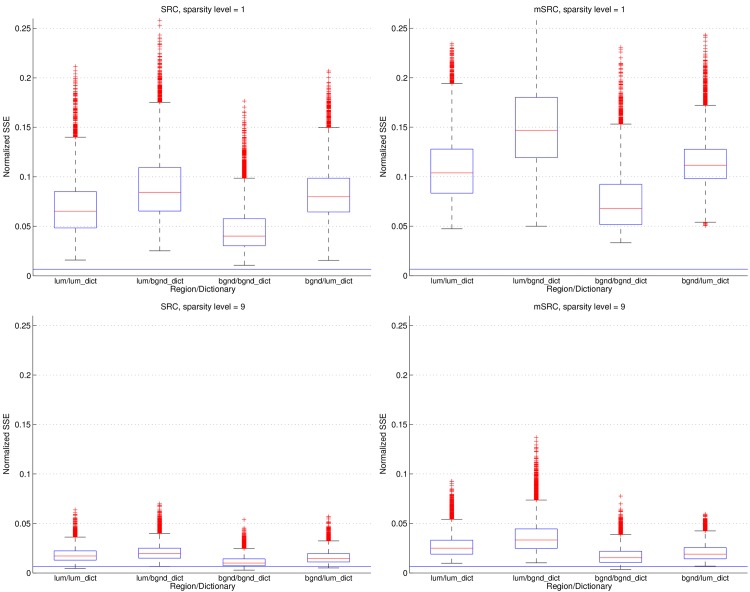
Normalized SSE errors of dictionary representations of 9 × 9 × 9 patches using sparsity levels 1 (top row) and 9 (bottom row) for a single representation (left column) versus 15 representations (right column, showing the average errors). The carotid lumen is denoted *lum* and the background is denoted *bgnd*. The noise level (see main text) is shown as a constant line in each plot. The results are shown as boxplots, where the blue box indicates the 25–75 percentile, the red bar indicates the median, the whiskers are determined by the default MATLAB boxplot settings (see MATLAB documentation), and the individual red plus-markers are outliers.

This effect of over-fitting was confirmed in an experiment illustrated in Fig 12. The plots show the sum of squared errors (SSE) (normalized by the sum of squares of the corresponding input signals) of the representations in a carotid lumen segmentation. In the plots the noise level is shown as a constant line in the bottom of each plot. The noise level is based on the standard deviation *σ* of image intensities in the lumen ROI:

$$\begin{array}{c} \text{noise}=\frac{1}{M}\sum_{i=1}^{M}\frac{N\sigma^{2}}{\sum_{j=1}^{N}x_{ij}^{2}}, \end{array}$$ (6)

where *M* is the number of feature vectors in the entire ROI, *N* is the dimensionality of the feature vectors, and *x*_*ij*_ denotes the *j*th element of the *i*th feature vector of the ROI. It is seen, that for a sparsity level of 9 and using only a single representation (i.e. SRC), both the correct and the incorrect dictionaries yield representation errors close to the noise level. When the representation error decreases, it becomes more influenced by the noise, as does the classification result. In mSRC, due to the slightly lower accuracy (higher normalized SSE) of the subsequent representations, the effect of over-fitting is reduced. We thus reason that the improvement of the results when using high sparsity levels and multiple representations are caused by a *joint effect* of statistical accumulation of evidence of class relationship *and* a reduction of over-fitting in subsequent representations.

### Effect of Dictionary Size

The dictionary size also provides flexibility in the representation. The larger (more overcomplete) the dictionary, the more sparsely a signal can be represented. The effect of dictionary size was tested for the carotid artery lumen segmentation and bifurcation detection experiments, see Figs 7 and 10. While the latter figure does not show a large impact of the dictionary size (or other parameters for that matter), in Fig 7 we notice at least one effect: in SRC (top row) the size of the dictionary has only a small influence on the performance, and increasing the sparsity level (as we discussed above) has a small negative effect. In mSRC (bottom row), however, the dictionary size has a pronounced positive impact on the results, although mainly for high sparsity levels.

The lower performance when using a small dictionary size and high sparsity in mSRC may be easily explained: in that case the proportion of atoms that are removed after each representation simply becomes too large (after 15 representations with a sparsity level of 9, a total of 135 atoms have been removed from the dictionary). The good performance of large dictionaries, on the other hand, may be explained by their higher redundancy: using larger dictionaries we can expect to accurately represent signals a larger number of times than with smaller dictionaries. According to this reasoning, a dictionary of 4000 atoms should perform better than one of 2000 atoms. This is, however, not the case in our experiments. The explanation may be the same as in the case of high sparsity levels: the redundancy provided by large dictionaries increases the chance of over-fitting.

### mSRC for Classification, Segmentation, and Detection

The mSRC method proposed in this paper performs well in all three tested settings and our hypothesis of improved performance over SRC has been confirmed. In the texture classification and carotid artery lumen segmentation experiments the effect of fusing the results of multiple representations is nicely reflected by the increasing hit-rates and Dice scores. The texture classification experiment presented a problem that was easily solved by mSRC as well as SRC and the two reference methods under the right parameter settings. However, mSRC did outperform SRC in the difficult case when a small patch size was used. As can be noted from Fig 4, the classification accuracy depends on the texture types. We hypothesize that the dominant vertical and horizontal structures of texture D21 are the reason why this texture are more easily distinguished from the more curvy structures of D28 and D77. The carotid artery lumen segmentation problem resembles the problems of prostate and hippocampus segmentation [21, 23]. The results of our experiments add to the evidence of the applicability of SRC methods in medical image analysis. In this application, we have seen the most clear example of the improvement of mSRC over SRC, and mSRC also outperformed the reference methods *K*-NN and SVM. Finally, in the bifurcation point detection experiments, the results of mSRC were reasonable (though not as good as for *K*-NN), but the results in Figs 9 and 10 looked somewhat erratic. Two tendencies are clear though. First, fusing results of three representations in mSRC consistently works better than SRC. Second, larger patches lead to more stable performance and apparently less dependence on the sparsity level. It is clear that further experiments are needed to investigate the effect of the mSRC parameters in this setting.

One important difference between the classification and segmentation experiments versus the point detection experiments is the huge difference in the statistical basis of the results. In the classification and segmentation settings, the hit-rate and Dice scores are based on thousands of voxelwise classifications, whereas in the point detection task a single voxel in a ROI of ∼ 100 000 voxels must be found and the median distance measure shown in the result figures is based on only 16 such detections (20 in the experiment on the test set). To properly test the effect of mSRC for bifurcation point detection, a much larger test set would be needed.

### mSRC versus *K*-NN

In all three experimental setups in this study, the performance of mSRC and *K*-NN are in the same range. One way to understand this, is to view mSRC as a generalization of *K*-NN. If the dictionary is simply the training set, the sparsity level is fixed to 1, and the single non-zero coefficient of each sparse coding vector is constrained to be equal to 1, then the two methods are exactly equivalent. In the texture experiments, *K*-NN performs particularly well (Fig 5). This experiment is an example of a situation where we do not gain discrimination power by the mSRC generalization, in particular, by compressing the training data into a dictionary and allowing linear combinations of multiple atoms.

While mSRC removes K-NN’s constraint on the sparse coding coefficients, which will be advantageous in settings such as lumination (or bias field) invariant object detection or texture classification, this essentially makes the method incapable of intensity-based discrimination. Since intensity is one of the most informative features in many types of images, including medical ones, it would be interesting to reintroduce this information in the classification, by reimposing constraints on the sparse coding coefficients or, possibly, by including the sparse coding vectors in the discrimination problem.

A final note on the computational complexities of the methods is in place here: The naive implementations of the OMP search of mSRC and the nearest neighbor search of *K*-NN have approximate complexities of 𝓞(*mT*) and 𝓞(*S*_*t*_), respectively, where *m* is the dictionary size, *T* is the target sparsity-level, and *S*_*t*_ is the number of samples in the training set. Typically *mT* is in the order of a few thousands while *S*_*t*_ is in the order of hundreds of thousands or even millions. Without improving the naive implementations, mSRC is thus far more efficient than K-NN classification, which may further motivate the use of mSRC.

## Conclusions

We have presented a novel and effective generalization of the basic SRC classification scheme. Whereas basic SRC uses the residuals of a single approximation per class for classification, the presented method fuses the residuals of multiple approximations, exploiting the statistical redundancy of the dictionaries. The resulting classifier yields higher classification accuracy than basic SRC. We have provided a thorough evaluation of the method and compared it with *K*-NN and SVM. Since our method generalizes SRC, any classification scheme that uses sparse representations and one or more overcomplete dictionaries for classification, should be able to leverage the mSRC idea.
