# Correction: Multiple Sparse Representations Classification

**DOI:** 10.1371/journal.pone.0136827

**Published:** 2015-08-24

**Authors:** Esben Plenge, Stefan Klein, Wiro J. Niessen, Erik Meijering

The second author’s name is spelled incorrectly. The correct name is: Stefan Klein. The correct citation is: Plenge E, Klein S, Niessen WJ, Meijering E (2015) Multiple Sparse Representations Classification. PLoS ONE 10(7): e0131968. doi:10.1371/journal.pone.0131968

## References

[1] Plenge E, Klein SS, Niessen WJ, Meijering E (2015) Multiple Sparse Representations Classification. PLoS ONE 10(7): e0131968 doi:10.1371/journal.pone.0131968 26177106 10.1371/journal.pone.0131968 PMC4503426

